# Embracing the heterogeneity of neural stem cells in the subventricular zone

**DOI:** 10.1016/j.stemcr.2025.102452

**Published:** 2025-03-20

**Authors:** Stefania Apostolou, Vanessa Donega

**Affiliations:** 1 Amsterdam UMC location Vrije Universiteit Amsterdam, department of Anatomy and Neurosciences, De Boelelaan 1117, Amsterdam, the Netherlands; 2 Amsterdam Neuroscience, Cellular and Molecular Mechanisms, Amsterdam, the Netherlands

**Keywords:** single-cell transcriptomics, adult subventricular zone, neural stem cells, quiescence, neural stem cell states, neural stem cell isolation

## Abstract

Neural stem cells (NSCs) of the subventricular zone (SVZ) could be a potential source for brain repair. These are heterogeneous cells with distinct activation states. To identify NSCs in the SVZ, different markers are used, including Gfap, Nestin, and Sox2. A comparison of these different methods to assess if the NSC marker used is selective toward specific NSC states is currently lacking. Here, we integrated six previously published single-cell RNA sequencing datasets from the adult mouse SVZ, where different methods were used to identify NSCs. Our data show that the approach used to isolate NSCs favors certain cell states over others. Our analyses underscore the importance of enriching for the NSC population of interest to increase data granularity. We also observed that cells with lower gene expression can be assigned incorrectly to clusters. We provide a framework for choosing the most optimal approach to enrich for NSC states of interest.

## Highlights

• The approach used for NSC isolation selects certain cell states over others
• Framework to select the most appropriate marker to enrich for specific NSC states
• Enriching for cells of interest increases data granularity
• Cells with lower gene expression can be assigned to clusters incorrectly

## Graphical abstract

**Figure:**
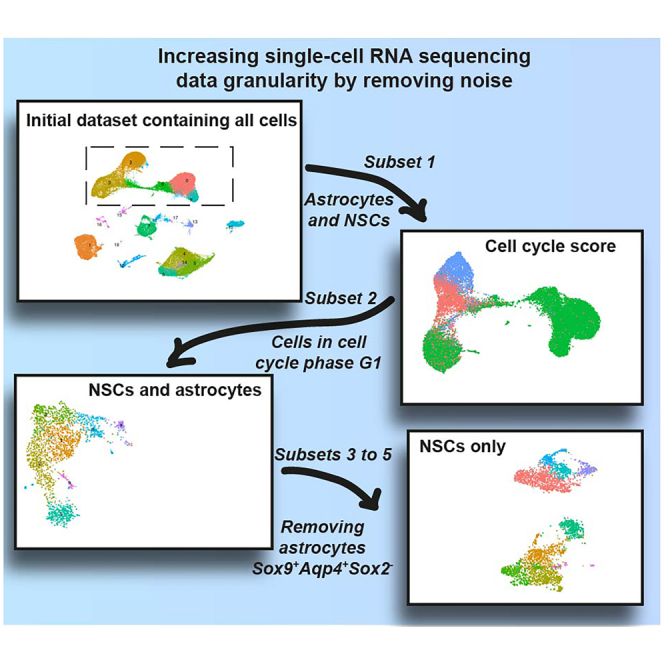

## Teaser

In this resource article, Apostolou and colleagues show that the approach used to isolate NSCs selects certain cell states over others. Our analyses underscore the importance of enriching for the NSC population of interest to increase data granularity. We provide a much-needed framework for choosing the most optimal approach to enrich for NSC states of interest.

## Introduction

Neural stem cells (NSCs) remain in two specific regions of the adult mammalian brain, including the subventricular zone (SVZ) that aligns the lateral ventricles. Neurogenesis continues in the SVZ during adulthood in most mammals (Chaker et al., 2016; Frisén, 2016). In rodents and non-human primates, new neurons generated in the SVZ migrate to the olfactory bulb. Most NSCs enter quiescence during brain development (Fuentealba et al., 2015; Furutachi et al., 2015) and, with aging, become increasingly quiescent (quiescent NSC [qNSC]) (Kalamakis et al., 2019; Leeman et al., 2018). This is defined as a non-proliferative state where NSCs usually exit the cell cycle and enter the G0 phase (Brunet et al., 2023; Fuentealba et al., 2015; Furutachi et al., 2015; Kalamakis et al., 2019; Leeman et al., 2018; Llorens-Bobadilla et al., 2015; Otsuki and Brand, 2018). Interestingly, the time point when NSCs enter quiescence depends on their spatial location within the SVZ with NSCs from the lateral wall entering quiescence embryonically (Fuentealba et al., 2015; Furutachi et al., 2015) and NSCs from the dorsal wall entering quiescence early postnatally (Borrett et al., 2020; Marcy et al., 2023). It is thought that this state is important to prevent NSC depletion and malignancy (Cheung and Rando, 2013). This quiescent state is reversible, and NSCs shift between an activated proliferative phase and quiescence. Single-cell RNA sequencing studies in NSCs from the mouse SVZ revealed that qNSCs go through intermediate activation states before becoming fully active entering a so-called primed-quiescent state (primed-quiescent NSCs [pqNSCs]) (Basak et al., 2018; Dulken et al., 2017; Llorens-Bobadilla et al., 2015). This shift from quiescence to activation is associated with gradual changes in, among others, metabolism, protein translation, and an increase in the expression of cell cycle genes (Basak et al., 2018; Dulken et al., 2017; Leeman et al., 2018; Llorens-Bobadilla et al., 2015; Shin et al., 2015). Understanding the dynamic NSC states and the mechanisms that regulate quiescence or activation could provide targets to stimulate NSC activation to promote brain repair following injury or in neurodegenerative diseases.

Single-cell RNA sequencing studies have identified genetic fingerprints for NSCs in different activation states (Basak et al., 2018; Belenguer et al., 2021; Codega et al., 2014; Dulken et al., 2017; Llorens-Bobadilla et al., 2015; Mizrak et al., 2019). Deep quiescent NSCs express *Id3* and *Aldoc* and lack the expression of *Acsl1*, *Egfr*, and *Fgfr3* that are associated with NSC activation, and their expression suggests a primed-quiescent state (Basak et al., 2018; Dulken et al., 2017; Llorens-Bobadilla et al., 2015; Shin et al., 2015; Trapnell et al., 2014). Active NSCs are defined by the expression of *Mki67* and *Mcm2* (Chaker et al., 2016; Dulken et al., 2017; Llorens-Bobadilla et al., 2015). RNA sequencing studies underline the heterogeneity among NSCs and capture a continuum of gradual changes in activation or quiescence states (Llorens-Bobadilla et al., 2015).

Different markers are used to identify NSCs in the mouse SVZ, including Gfap, Nestin, and Sox2 (Pastrana et al., 2009). Reporter mouse lines for cells that express Gfap and Nestin are commonly used to isolate NSCs for single-cell RNA sequencing. Another method that is used combines a reporter mouse line with a fluorescent-activated cell sorting (FACS) purification step for cells positive for Prominin1 (Prom1) and negative for Egfr (Dulken et al., 2017; Marques-Torrejon et al., 2021) as putative qNSCs. These studies provided important insights into the biology of adult NSCs and the quiescence and activation continuum. However, a comparison of these different approaches to determine whether the marker used to detect NSCs is selective toward certain NSC states is lacking. This could generate a much needed framework for selecting the most appropriate markers to enrich for specific NSC states of interest and avoid bias when interpreting the dynamic behavior of NSCs, their biology, and heterogeneity. Here, we integrated six previously published single-cell RNA sequencing datasets from the adult mouse SVZ, where different approaches were used to identify NSCs (Dulken et al., 2017; Hamed et al., 2022; Kalamakis et al., 2019; Mizrak et al., 2020; Xie et al., 2020). Our analyses show that the approach used for NSC isolation selects for certain cell states over others. Furthermore, our analyses highlight that enriching for the NSC population of interest increases data granularity and that cells with lower gene expression can be assigned to clusters incorrectly. We provide a framework for choosing reporter mouse lines that best reflect the NSC states of interest.

## Results

### Subsetting clusters to remove noise

**Figure 1:**
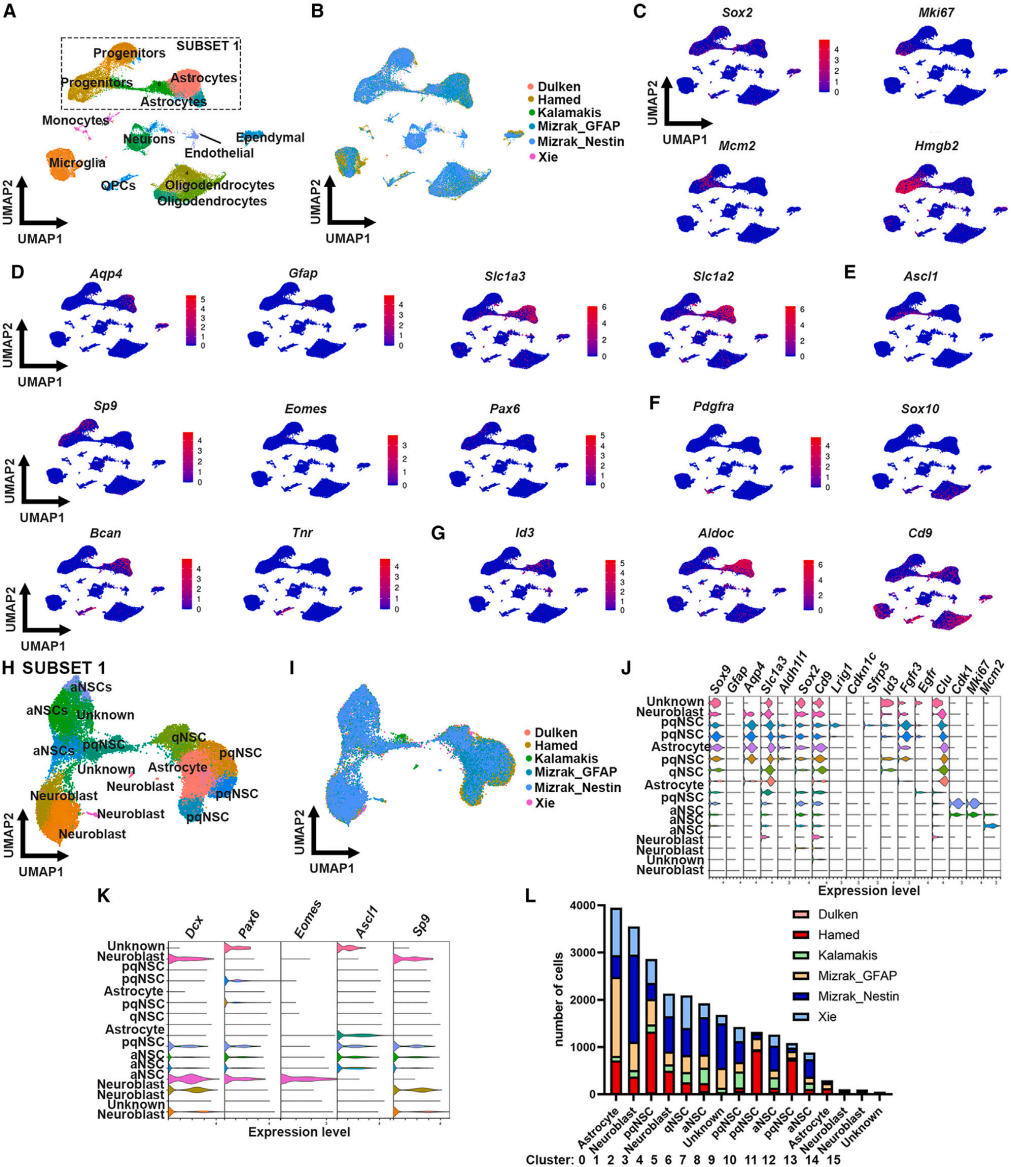
Subsetting to identify cell populations and cell states of interest (A and B) UMAP projection of cell clusters shown by cluster (A) and study (B). Insert highlights the clusters for subsetting. (C–G) Feature plots for a selected number of canonical markers for NSCs, proliferation, progenitors (C), astrocytes (D), GABAergic and glutamatergic progenitors (E), oligodendrocyte progenitor cells (OPCs) and oligodendrocytes (F), and quiescence (G). (H and I) Subset of the data in (A) showing the UMAP projection of astrocyte, progenitors, and NSCs shown by cluster (H) and study (I). A total of 24,729 cells remained after subsetting. (J and K) Violin plots showing the expression of astrocyte, NSC, quiescence, activation gene markers (J) and neuroblasts, and GABAergic and glutamatergic progenitor gene markers (K). (L) Number of cells per cluster (cell subtype and cell state) and per study.

To determine whether different approaches to isolate NSCs of the mouse SVZ could enrich for different NSC states, we integrated six single-cell RNA sequencing datasets from the mouse SVZ. We selected single-cell RNA sequencing datasets from mice between 39 and 90 days old (Table S1), where either reporter mouse lines were used for Gfap, Nestin, or Sox2 (Hamed et al., 2022; Mizrak et al., 2020; Xie et al., 2020), and an FACS-based approach for Gfap and Prom1-positive cells (Dulken et al., 2017; Kalamakis et al., 2019). We performed unbiased cluster analysis using the Louvain algorithm and uniform manifold approximation and projection (UMAP) (Butler et al., 2018; Hashimshony et al., 2016), identifying several cell populations including oligodendrocytes, microglia, and ependymal cells (Figures 1A–1G and S1). We identified the clusters that contained progenitors and astrocytes for subsetting (insert Figure 1A). After running ScaleData and unbiased cluster analysis on these cells (see also methods), subset 1 was generated (Figure 1H), identifying 16 clusters, including not only NSCs but also niche astrocytes (*Sox9*^*+*^*Aqp4*^*+*^*Sox2*^*−*^) and neuroblasts (*Dcx*^+^) (Figures 1J and 1K). The stem cell clusters showed NSCs in different states from activation to quiescence (Figures 1J and 1K). Active NSCs expressed proliferation markers *Mki67* and/or *Mcm2.* Distinguishing qNSCs from pqNSCs was based on the expression of *Egfr*, which, if present, suggested a primed-quiescent state. All datasets contributed to the different cell subtypes and cell states (Figure 1L). pqNSCs were the largest NSC state identified in subset 1 with a total of 6,694 cells, followed by active NSC with 4,066 cells and qNSCs with 2,092 cells (Figure 1L).

### Disentangling qNSCs from niche astrocytes

**Figure 2:**
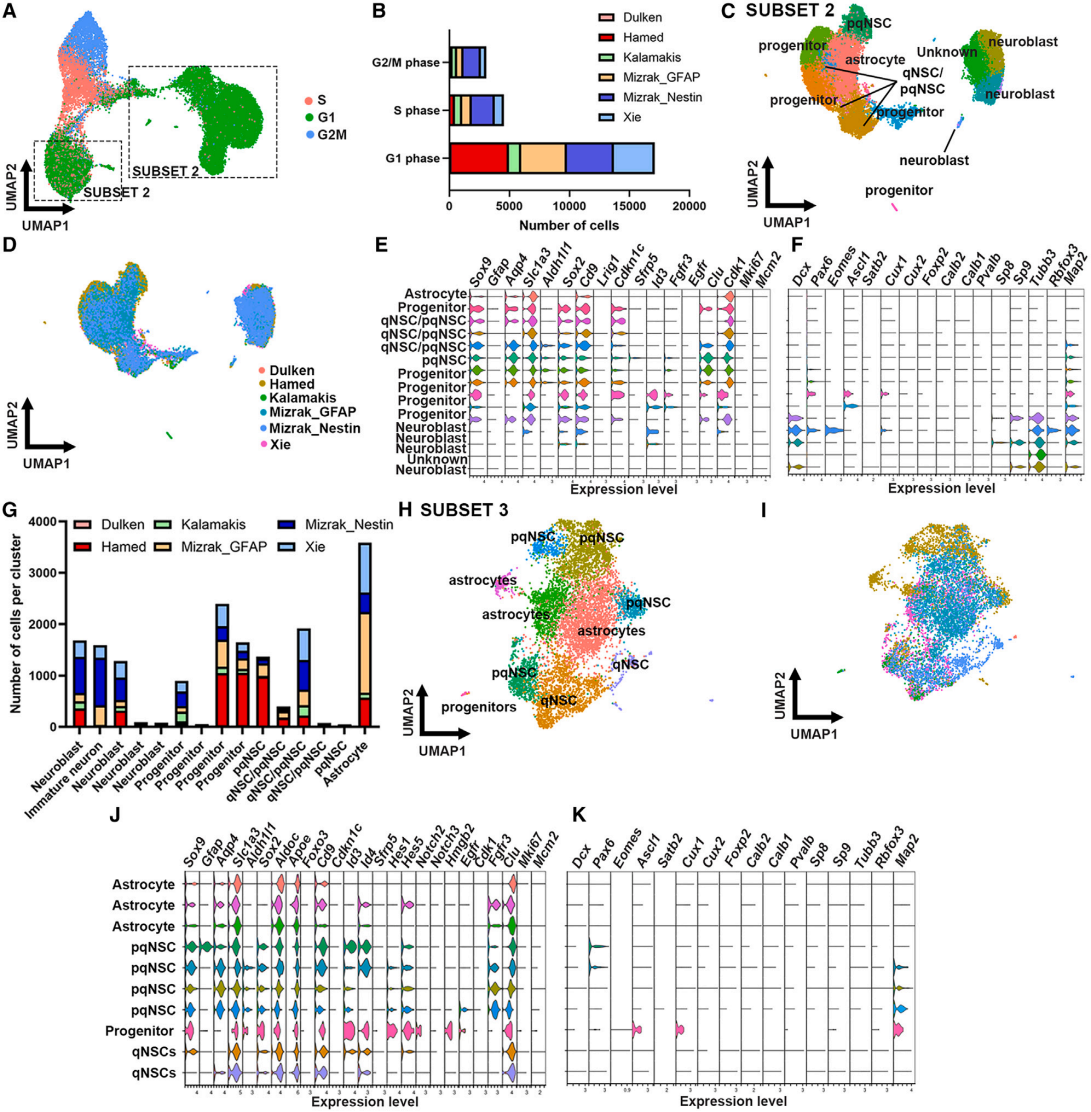
Identifying the different NSC cell states (A) UMAP projection of cell clusters shown by cell cycle scores. Insert highlights the cluster for subsetting. (B) Number of cells per cell cycle phase for each study. (C and D) Subset of the data in (A) showing the UMAP projection of cell clusters shown by cluster (C) and study (D). (E and F) Violin plots showing the expression of astrocyte, NSC, quiescence, activation gene markers (E) and neuroblasts, GABAergic and glutamatergic progenitors, and neuronal gene markers (F). (G) Number of cells per cluster (cell subtype and cell state) and per study. (H and I) UMAP projection of cell clusters shown by cluster (H) and study (I). (J and L) Violin plots showing the expression of astrocyte, NSC, quiescence, activation gene markers (J) and neuroblasts, GABAergic and glutamatergic progenitors, and neuronal gene markers (L).

It is difficult to distinguish niche astrocytes from qNSCs because they share many canonical markers (Arellano et al., 2021; Cebrian-Silla et al., 2021; Dulken et al., 2019; Zywitza et al., 2018). However, by de-noising the data, that is, removing cell types that are not of interest to the study, one can increase the resolution and the power to focus on smaller differences in gene expression. As we are interested in the NSC population and its different cell states, we next performed cell cycle analysis using the cell cycle regression pipeline from Seurat (Tirosh et al., 2016). This confirmed the cell subtypes and cell states identified in subset 1 (Figure 1H), with activated NSCs (aNSCs) in the G2/M and S phases and the remaining cells in the G1 phase (Figure 2A; Table S2). Hamed et al. (Sox2 reporter mice), Mizrak et al. (Gfap reporter mice), and Xie et al. (Nestin reporter mice) had the largest proportion of cells in the G1 phase, while Kalamakis et al. (Gfap^+^Prom1^+^ cells) and Mizrak et al. (Nestin reporter mice) had relatively similar proportions of cells in the different cell cycle phases (Figure 2B). We next subsetted the G1 cluster to further differentiate the niche astrocytes from qNSCs and pqNSCs generating subset 2 (Figures 2C and 2D). This revealed one niche astrocyte cluster (*Sox9*^*+*^*Aqp4*^*+*^*Sox2*^*−*^), four neuroblast clusters (*Dcx*^+^), four progenitor clusters (*Sox2*^*+*^*Cd9*^*+*^*Hmgb2*^*+*^ and *Pax6/Eomes/Ascl1*^*+*^), one pqNSC cluster (*Sfrp5*^*+*^*Egfr*^*+*^*Fgfr3*^*+*^), and three clusters that were ambiguous and could either be qNSCs or pqNSCs (Figures 2E and 2F). All datasets contributed to the different cell populations and cell states (Figure 2G).

**Figure 3:**
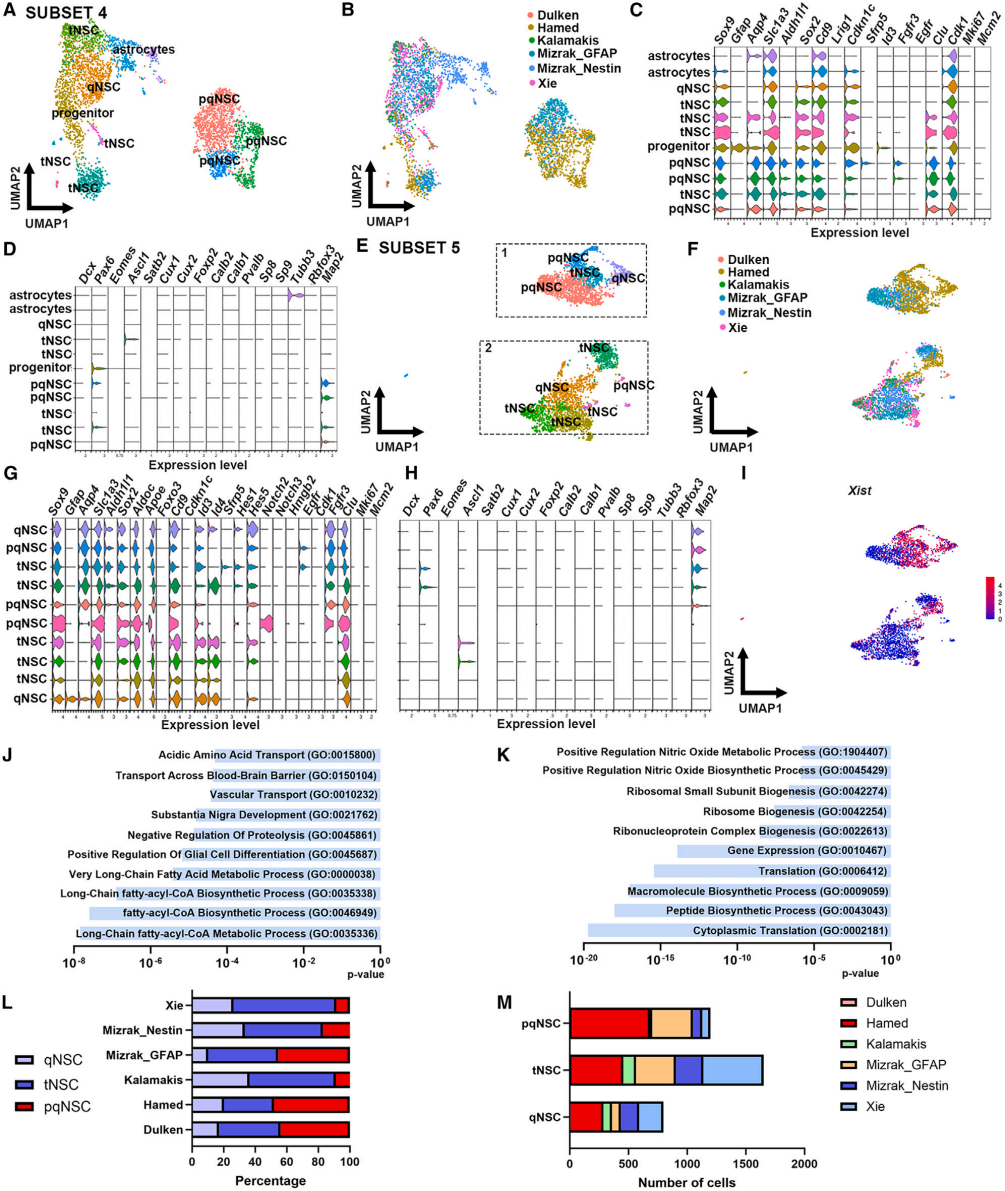
Proportions of NSC states per dataset (A and B) Subset of the data in (H) from Figure 2, showing the UMAP projection of cell clusters shown by cluster (A) and study (B). (C and D) Violin plots showing the expression of astrocyte, NSC, quiescence, activation gene markers (C) and neuroblasts, GABAergic and glutamatergic progenitors, and neuronal gene markers (D). (E and F) Subset of the data in (A) showing the UMAP projection of cell clusters shown by cluster (E) and study (F). (G and H) Violin plots showing the expression of astrocyte, NSC, quiescence, activation gene markers (G) and neuroblasts, GABAergic and glutamatergic progenitor, and neuronal gene markers (H). (I) Feature plot showing the expression of the sex gene *Xist*. (J–K) Graphs showing gene ontology analysis of biological processes for cluster 1 (J) and cluster 2 (K). (L) Percentage of cells per cell state and study. (M) Number of cells per cell state and study.

We next subsetted the dataset to remove the niche astrocytes, neuroblasts, and progenitor cells. After running ScaleData and unbiased cluster analysis on the pqNSCs, qNSCs/pqNSCs, and unknown cells (see also methods), subset 3 was generated identifying 10 clusters including three niche astrocytes (*Sox9*^*+*^*Aqp4*^*+*^*Sox2*^*−*^) and one progenitor cluster (*Sox2*^*+*^*Cd9*^*+*^*Hmgb2*^*+*^*Ascl1*^*+*^*Cux1*^*+*^) (Figure 2H) despite having removed those cell types when subsetting the data from subset 2 (Figure 2C). This suggests that when dealing with two cell types that are very similar in gene expression, and when within one cell type there is a subpopulation that has lower expression of some key defining genes for this cell type, it could end up being wrongly assigned to a different cluster. Most niche astrocytes were identified in the Mizrak_GFAP dataset (Figures 2H and 2I). Two clusters corresponded to qNSCs (*Sox2*^*+*^*Id3/Id4*^*+*^*Hes1/Hes5*^*+*^*Egfr*^*-*^*Fgfr3*^*−*^), and four clusters were identified as pqNSC (*Egfr*^*+*^*/Fgfr3*^*+*^) (Figures 2J–2L). This round of subsetting identified a *Gfap*-expressing cluster that was absent from subsets 1 and 2 (Figure 2J). As it also expressed *Sox2* and *Fgfr3*, we classified this cluster as pqNSCs. Two additional rounds of subsetting were needed to identify and remove all niche astrocytes and progenitor cells, leaving only the NSC population of interest. This generated subset 4 (Figures 3A–3D), which contained two astrocyte clusters that were excluded from the dataset through subsetting. After running ScaleData and unbiased cluster analysis, subset 5 was generated (Figures 3E–3H). This pool of cells contained exclusively NSCs in different states of quiescence (Figures 3E–3H).

### Quiescence: A highly dynamic cell state

The final round of subsetting, generating subset 5, revealed 10 clusters, including two qNSC clusters (*Aqp4*^*+*^*Sox2*^*+*^*Hes1/Hes5*^*+*^*Id4*^*+*^) and three pqNSC clusters (*Id4*^*−*^*Fgfr3*^*+*^*Egfr*^*+/−*^*Notch2*^*+/−*^) (Figures 3G and 3H). As described previously, qNSCs activate gradually going through intermediate activation states (Dulken et al., 2017; Llorens-Bobadilla et al., 2015; Shin et al., 2015). This is reflected at gene expression level, with NSCs transitioning between quiescence and primed-quiescence, and primed-quiescence and activation. We identified five clusters as transitioning between states (referred to as tNSCs). Three clusters seemed to be transitioning from pqNSC to qNSC state as the NSCs expressed *Ascl1*, and quiescence markers *Id4* and *Hes5*, but lacked expression of *Aqp4*, a marker associated with qNSCs (Urban et al., 2019). Another two clusters could correspond to NSCs transitioning from primed-quiescence to activation as they expressed *Aqp4* and other quiescence markers such as *Hes1/Hes5*, primed-quiescence marker *Fgfr3* and primed-quiescence/activation marker *Egfr*, and progenitor marker *Pax6* (Figures 3G and 3H; Table S5)*.* Analysis of cluster-identifying markers (Table S3) identified some subcluster-specific gene expression; for example, subclusters of qNSCs and pqNSCs had higher expression of eight mitochondrial genes, a subcluster of pqNSCs showed higher *Notch2* expression, while a subcluster of tNSCs showed enrichment for *Hopx*. These differences in gene expression were too restricted for Gene Ontology (GO) analysis. It does, however, underlie the gain in increasing the data granularity by enriching for the NSC population of interest.

These 10 clusters were organized into two larger clusters that we named cluster 1 and cluster 2 (Figure 3E). Both clusters 1 and 2 contained NSCs in quiescent, transient, and primed-quiescent cell states (Figure 3E). As this distribution pattern did not correlate to the original study (Figure 3F), we determined whether sex differences could be driving the organization of these clusters. Indeed, previous studies showed sex differences in lineage potency of progenitors from the mouse SVZ (Mizrak et al., 2019) and the existence of pregnancy-associated SVZ domains, which are under homeostatic conditions more quiescent, but become neurogenic at specific moments during pregnancy (Chaker et al., 2023). Furthermore, NSC activation was shown to increase during pregnancy, which is mostly hormonally driven (Shingo et al., 2003). As information on the sex of mice used in the experiments was not available for all studies, we checked for the expression of *Xist*, a gene that is expressed exclusively in females. Both sexes were represented in both clusters 1 and 2 (Figure 3I), and therefore do not explain clusters 1 and 2. To determine whether the cells in these two clusters corresponded to different subpopulations of qNSCs, tNSCs, or pqNSCs, we performed GO analysis on the cluster-identifying markers of both clusters 1 and 2. This showed, for cluster 1, an enrichment for genes involved in fatty acid metabolism (e.g., fatty acid metabolic process) (Figure 3J), while cluster 2 showed enrichment for genes involved in protein translation (e.g., ribosome biogenesis) (Figure 3L). This suggested that the intrinsic cell properties drive the formation of clusters 1 and 2. All datasets contributed to the different cell states (Figure 3M). Most pqNSCs came from Hamed et al. and Mizrak et al. (Gfap reporter mouse). Sox2 reporter mouse line (Hamed et al.) returns mainly pqNSCs and around 20% qNSCs, while the Nestin reporter mouse line from both Mizrak et al. and Xie et al. and Gfap reporter mouse line with FACS for Prom1 from Kalamakis et al. yielded mostly tNSCs. Proportions of qNSCs varied from 10% (GFAP reporter mouse line, Mizrak et al.) to 36% (Kalamakis et al.) (Figures 3M and 3N).

**Figure 4:**
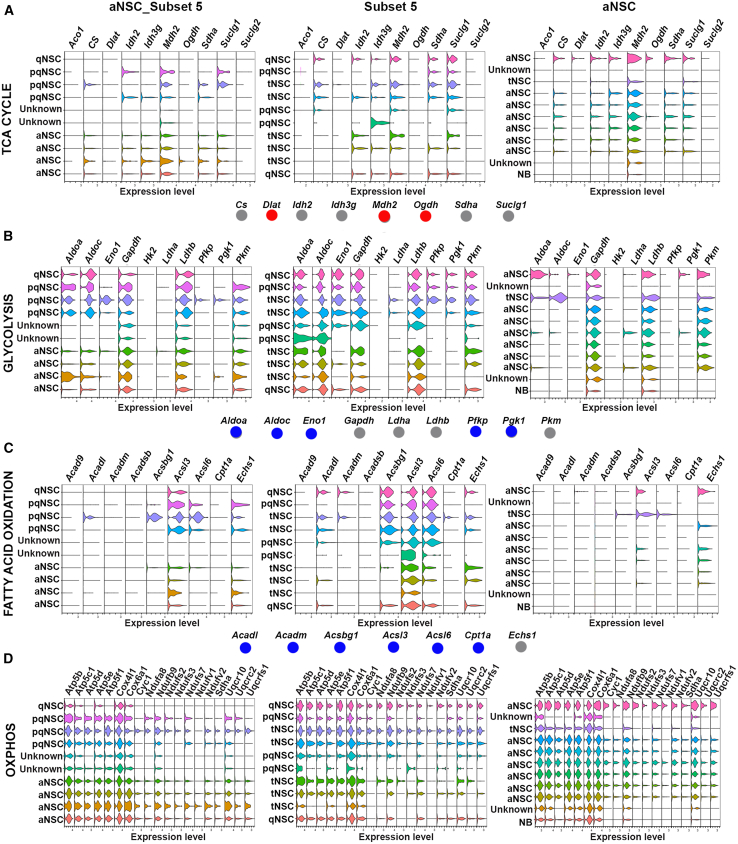
Metabolic pathways in different NSC states Violin plots showing the expression of genes involved in TCA cycle (A), glycolysis (B), fatty acid oxidation (C), and oxidative phosphorylation (D) for the aNSC_Subset 5 dataset (containing aNSCs, qNSCs, tNSC, and pqNSCs), and subset 5 (containing qNSC, tNSC, and pqNSC) and aNSCs (containing mainly aNSCs) datasets separately. Gene panels are based on the study by Scandella V et al. (2023). Dots below violin plots indicate higher expression in either aNSCs or qNSCs. A gray dot indicates that the gene is expressed in both NSC states, a blue dot means that the gene is highly expressed in qNSC, and a red dot indicates that the gene is highly expressed in aNSCs.

Fatty acid β-oxidation (FAO) is one of the main metabolic pathways where ATP is generated from the oxidation of fatty acids in the mitochondria. It was thought that qNSCs were in a low energy demanding state and relied mostly on glycolysis and FAO (Beckervordersandforth, 2017; Llorens-Bobadilla et al., 2015; Shin et al., 2015), and as the cell became active and differentiated, their metabolism shifted toward oxidative phosphorylation (Chaker et al., 2016; Cheung and Rando, 2013; Llorens-Bobadilla et al., 2015; Lunt and Vander Heiden, 2011). However, recent studies suggest that qNSCs are actually in an active rather than low metabolic state and that their metabolic phenotype is more complex than originally proposed (Knobloch et al., 2013; Petrelli et al., 2023; Scandella et al., 2023; Wani et al., 2022). Here, we used the metabolic gene panels from the study by Scandella et al. (2023), to further clarify the role of metabolic pathways in different NSC states. To include NSCs in the active state to the analysis, the cells in the G2M and S phases (containing neuroblasts and aNSCs) that were removed from the dataset following cell cycle analysis (Figures 2A–2C) were integrated to subset 5 generating the aNSCs_Subset 5 dataset, which contained aNSCs, tNSCs, pqNSCs, and qNSCs. By viewing gene expression not only in the aNSC_Subset 5 dataset but also in both datasets separately, that is in the aNSCs dataset (Figure S2) and subset 5 dataset, clusters with low gene expression could be detected. While in the aNSC_Subset 5 dataset, the qNSC population does not express any of the genes involved in the tricarboxylic acid (TCA) cycle, when looking at the subset 5 dataset only, both qNSC clusters express several genes involved in the TCA cycle (Figure 4A). As shown in the study by Scandella et al. (2023), we also observed a mixed gene expression pattern between NSC states and at the subcluster level. For example, the gene *Idh2*, involved in the TCA cycle, was expressed in three out of five pqNSC clusters (Figure 4A). Most genes from the TCA cycle and oxidative phosphorylation metabolic pathways were expressed in NSCs in different states, with variability between subclusters within the NSC state (Figures 4A–4D). An exception to this was FAO that was mostly expressed in qNSCs and pqNSCs compared to aNSCs (Figure 4C) and glycolysis, which was also more strongly expressed in qNSCs/pqNSCs (Figure 4B). In contrast to the study by Scandella et al. (2023), where glycolysis genes *Hk2* and *Ldha* were increased in aNSCs, our data showed low expression in all NSC states (Figure 4B). Oxidative phosphorylation was strongly expressed in all NSC states (Figure 4D). Our analyses further underscore the heterogeneity and dynamic nature of NSC states and the importance of analyzing gene expression at both metabolic pathway and cluster levels.

## Discussion

The rise of single cell-RNA sequencing techniques allowed us to study NSCs of the mouse SVZ with unprecedented resolution. The single-cell resolution of the technique ignited a new era in biology with the identification of different cell (sub)types and cell states. We gained a better understanding of the different NSC states and their heterogeneity. Despite having identified gene signatures specific for different NSC states, we have not yet reached a consensus on which markers to use to identify specific NSC states. Different markers are used to identify NSCs from the mouse SVZ, yet an unbiased comparison of the proportions of NSCs that are isolated in different states by different approaches is still lacking. Here, we compared six previously published datasets (Dulken et al., 2017; Hamed et al., 2022; Kalamakis et al., 2019; Mizrak et al., 2020; Xie et al., 2020) that used four common approaches to isolate NSCs of the mouse SVZ. Our analysis provides a framework to guide new studies in selecting the NSC marker that enriches for the NSC state(s) of interest.

Previous studies showed that qNSCs and pqNSCs have lower and sparser gene expression than other cell types including oligodendrocytes and niche astrocytes (Tosoni et al., 2023). These cell states will be clouded when in a large dataset containing different cell types. Cells are clustered based on gene expression, and therefore, when in a noisy environment, genes that are highly expressed will have the upper hand in determining the position of the cell within the 2D UMAP space. After removing the noisy cells, i.e., the cells with many highly expressed genes, more resolution is gained enabling the identification of different cell states. Genes that were at first not visible due to their low expression can now be identified in specific subclusters. One example of this is the expression of *Gfap*, which was only detected at a later stage after the second round of subsetting, and only in a few subclusters. We showed that niche astrocytes can be distinguished from qNSCs by their lack of *Sox2* expression. A previous study in 4 months old adult mice showed that niche astrocytes are enriched for *Clmn*, *Atp13a4*, *Eps8*, *Pcdh7*, and *Syne1* compared to qNSC/pqNSCs (Cebrian-Silla et al., 2021). Thus, through the combination of several markers, it is possible to distinguish niche astrocytes from qNSCs in the adult SVZ (Table S5).

All four approaches to isolate stem cells yielded NSCs in diverse states albeit in different proportions. This is important to keep in mind when designing a study. In subset 1, which contained astrocytes, neuroblasts, progenitors, and active NSCs (Figure 1H), less than 2% of the cells were in a quiescent state, with the exception of Dulken, where almost 10% of the cells were pqNSC/qNSC. Both Dulken and Kalamakis yielded the highest percentage of aNSCs (34%–38%). The other more high-throughput studies showed lower percentages of aNSCs of around 6 to 22%.

Another important aspect to consider when designing a study is the SVZ region where the cells will be isolated from. The NSCs from the SVZ are highly heterogeneous and show regional differences in activation and lineage potential (Azim et al., 2015; Chaker et al., 2016; Kawai et al., 2017; Mizrak et al., 2019). The dorsal SVZ gives rise to glutamatergic and GABAergic neurons, and oligodendrocytes, while the lateral and medial walls give rise to GABAergic neurons and oligodendrocytes (Azim et al., 2016; Brill et al., 2009; Donega and Raineteau, 2017; Fiorelli et al., 2015; Merkle et al., 2007, 2014; Winpenny et al., 2011). NSCs from the medial wall are more quiescent than the lateral and dorsal walls (Barazzuol et al., 2017; Benito et al., 2018; Fiorelli et al., 2015). While Hamed et al. isolated cells from all the SVZ walls, the other studies focused on one or two of the SVZ walls (Table S1). In the Mizrak et al. datasets where NSCs were isolated from both the medial and lateral SVZ walls using different NSC markers, our analysis showed that the Mizrak_Nestin dataset had a higher percentage of tNSCs of 49.7%, while Mizrak_Gfap equal amounts of tNSCs (44.5%) and pqNSCs (45.3%). Interestingly, the Xie et al. dataset, where a Nestin reporter mouse was used and NSCs were isolated from both the dorsal and lateral SVZ walls, showed a higher proportion of tNSCs (65.2%) as well. This suggests that Nestin is more highly expressed in tNSCs and that the Nestin reporter mouse line could be used to enrich for tNSC.

Once qNSCs, pqNSCs, and tNSCs remained in the dataset, we were able to increase data granularity and detect different subpopulations within NSC states. Interestingly, subset 5 showed that similar cell states clustered based on metabolic state and protein translation, suggesting potential differences in their capacity to activate. Our assessment of metabolic pathways highlighted the importance of analyzing gene expression at both metabolic pathway and cluster levels. Our analysis of metabolic pathway gene expression showed that while glycolysis and FAO were mainly expressed in qNSC/pqNSCs, these cells also expressed markers from the oxidative phosphorylation and TCA cycle metabolic pathways. This is in agreement with growing evidence that qNSCs are in an active rather than low metabolic state (Knobloch et al., 2013; Petrelli et al., 2023; Scandella et al., 2023; Wani et al., 2022). Our data also demonstrate that genes from the FAO and glycolysis pathways can be used to identify qNSC/pqNSCs in combination with other quiescence and NSC markers.

Our data confirm previous work (Chaker et al., 2016; Llorens-Bobadilla and Martin-Villalba, 2017; Llorens-Bobadilla et al., 2015; Urban et al., 2019) showing that multiple markers are necessary to accurately detect different NSC states. The difficulty in finding specific markers and gene signature for specific cell states could be a consequence of NSCs being in a gradient of NSCs states when progressing from quiescence to activation and when exiting the cell cycle and entering quiescence. Increasing the number of NSCs in the dataset and enriching for the population of interest could help to denoise and refine the data. Altogether, our data further underscore the heterogeneity and dynamic nature of NSCs (Table S5) (Kalinina and Lagace, 2022; Llorens-Bobadilla et al., 2015).

Here, we focused on single-cell RNA sequencing datasets from young mice. It would be of interest to determine if using similar approaches in older mice, where qNSCs are more abundant, would reveal similar specificity of NSC markers to identify different cell states. Moreover, previous work has shown that the gene expression and gene signature of qNSCs changes during aging (Brunet et al., 2023; Kalamakis et al., 2019; Leeman et al., 2018; Llorens-Bobadilla and Martin-Villalba, 2017). We used several markers to identify different NSC states based on previous work; however, the use of these specific markers could introduce bias and limit the identification of new gene signatures. In the same way that we observed that astrocytes were incorrectly assigned to NSC clusters, NSCs might also be misassigned to astrocyte clusters and, therefore, be excluded from further analysis. Hence, the final NSC cluster could be downsampling the NSC pool. Another important limitation is that we cannot take into consideration possible (and likely) differences in isolation efficiency between different studies, which not only affect cell survival but could also affect NSC state.

Understanding the dynamic NSC states and the mechanisms that regulate quiescence or activation could provide targets to stimulate NSC activation to promote brain regeneration following injury or in neurodegenerative diseases. It could also help to better understand how NSCs in different cell states are affected by injury or by disease pathology in neurodegenerative diseases, which could provide novel targets to boost the capacity of the brain to regenerate. We show that to identify rare cell states within a continuum, not only is the isolation approach relevant, but it is also important to remove competing larger cell populations or cell states that may otherwise cloud the rare cell state of interest. This work provides a framework to choose the most appropriate isolation method to enrich for NSC states of interest.

## Methods

### Dataset selection

We selected previously published single-cell RNA sequencing datasets of mouse SVZ NSCs that used different common NSC markers for their isolation. We excluded single-nucleus RNA sequencing datasets to avoid confounding factors relating to potential differences in nuclear and cytoplasmic RNA profiles and levels. Next, we decided to focus on datasets from young adult mice no older than 90 days, as aging is known to affect NSC number and cell states. Datasets from early postnatal mice (<20 days) were also excluded to limit the difference in age between datasets to reduce potential age effect in the analysis. We included datasets from both male and female mice as hormonal differences are also known to affect NSC behavior (Chaker et al., 2023; Mizrak et al., 2019; Shingo et al., 2003). This resulted in six single-cell RNA sequencing datasets for analysis.

### Filtering and normalization of single-cell RNA sequencing datasets

We integrated six single-cell RNA sequencing datasets from the adult mouse SVZ aged 39 to 90 days old from both female and male mice. These single-cell RNA sequencing datasets were selected based on the cell isolation method used. We included datasets that used reporter mouse lines for Gfap, Nestin, or Sox2 (Hamed et al., 2022; Mizrak et al., 2020; Xie et al., 2020) and an FACS-based approach for Gfap and Prom1-positive cells (Dulken et al., 2017; Kalamakis et al., 2019). Quality check and filtering was performed on R Studio v.4.4.1 and Seurat v.4.3.0 (Butler et al., 2018) using the following parameters: to create the Seurat Object only genes that were detected in at least three cells and cells that expressed at least 200 genes were kept. Cells that had less than 200 genes or more than 7,500 genes detected were filtered out. Normalization was done using Seurat (NormalizeData function with LogNormalize method) where a generalized linear model for each gene is constructed (Hafemeister and Satija, 2019). Scaling was performed to remove the effect on the normalized expression values of differences in sequencing depth, and library preparation with the ScaleData function.

### Integration of six single-cell RNA sequencing datasets

Data integration was performed with Seurat v.4.3.0 as described in the study by Stuart et al. (2019). This pipeline enables the integration of multiple datasets, which cluster by cell type instead of platform technology or species. The data integration is done by first identifying in each dataset the 2,000 most variable genes using the FindVariableGenes function. It postulates that if the datasets to be integrated share similarities and if a subset of cells have a shared biological state, a set of molecular features (anchors) could be identified. These anchors were identified with the FindIntegrationAnchors function with dims set to 30. A Seurat Object is created with the IntegratedData function, which passes the identified anchors to the Seurat Object. This resulted in a Seurat Object of 53,116 cells and 32,817 genes.

### Single-cell clustering and visualization

Following integration, the top 35 principle components (PCs) were used to identify clusters (using RunPCA, FindNeighbors, and FindClusters functions with the resolution set to 0.4). For visualization of the clusters, UMAP coordinates were calculated in PCA space using Seurat (RunUMAP function) with PC set at 35. UMAP plots were then colored by cluster identity, study, or gene expression values (FeaturePlot function). Marker genes were determined using the FindMarkers function. Genes were considered marker genes when they were expressed in more than 60% of the cells with an adjusted *p* value of less than 0.01. The Wilcoxon rank-sum test was used to identify marker genes. *p* value adjustment was performed using the Bonferroni correction. Single cells were clustered based on their cell cycle score using the cell cycle regression score pipeline from Seurat. Subsetting data were performed by selecting the clusters of interest and performing ScaleData. Clusters were visualized by calculating UMAP (with reduction set to PCA) coordinates following identification of clusters with the number of PCs set to 30 (RunPCA, FindNeighbors, and FindClusters function) with the resolution set to 0.8 or 0.5. See also Figure S3 for a schematic overview of the workflow to subset the data. Violin plots (VlnPlots function), which show normalized counts, were used to visualize and analyze the data.

### GO enrichment analysis

GO *biological process* analyses were performed on 212 differentially expressed genes (150 upregulated genes and 62 downregulated genes) with an adjusted *p* value <0.01 and a pct of at least 0.5 in one of the two groups that were identified after comparison of cluster 1 with cluster 2 from subset 5 (Table S4). The GO *biological process* from the EnrichR web-based tool (Chen et al., 2013; Kuleshov et al., 2016) was used (http://amp.pharm.mssm.edu/Enrichr/). Graphs were created in GraphPad Prism 7 (version 9.5.1, La Jolla, Ca, USA).

## Resource availability

### Lead contact

Further information and requests for resources should be directed to the lead contact, Vanessa Donega (v.donega@amsterdamumc.nl).

### Materials availability

This study did not generate new material.

### Data and code availability

All RNA sequencing datasets used in this study can be found on GEO through their respective bioproject or accession numbers (PRJNA324289 (Dulken et al., 2017), GSE200202 (Hamed et al., 2022), GSE115626 (Kalamakis et al., 2019), GSE134918 (Mizrak et al., 2020), and GSE107220 (Xie et al., 2020)). The accession number for the R script used to analyze the data and the RDS objects reported in this paper can be found on Mendeley Data (“Analysis scRNA-seq mouse SVZ” https://doi.org/10.17632/mwmbrfz6sd.1).

## Acknowledgments

This study was supported by the Startergrant from the 10.13039/100019573Amsterdam UMC to V.D.

## Author contributions

S.A. and V.D. performed scRNA-seq analyses. V.D. supervised data analyses. V.D. wrote the manuscript with input from all of the authors.

## Declaration of interests

The authors declare no competing interests.

## Supplemental information

Document S1. Figures S1–S3 and Tables S1 and S5

Table S2. S score and G2_M score per cell calculated with the cell cycle scoring analysis script from the Seurat pipeline

Table S3. List of differentially expressed genes for each of the clusters in Figure 3E Differential gene expression was tested with Bonferroni corrected Wilcoxon rank-sum test. Genes were considered significant when adjusted P-value <0.01. Genes were considered cluster marker genes when adjusted P-value <0.01, and it was lowly expressed in one of the two groups (pct2 < 0.5), while being expressed in more than half of the cells in the other group (pct1 > 0.5). pct = the percentage of cells where the feature gene is detected in the first or second group.

Table S4. List of differentially expressed genes between cluster 1 and cluster 2 Differential gene expression was tested with Bonferroni corrected Wilcoxon rank-sum test. Genes were considered significant when adjusted P-value <0.01.

Document S2. Article plus supplemental information
