# Supplementary material for: Embracing the heterogeneity of neural stem cells in the subventricular zone
Source: Stem Cell Reports. 2025 Mar 20;20(9):102452. doi: 10.1016/j.stemcr.2025.102452 (PMC12447326; doi:10.1016/j.stemcr.2025.102452)
Supplement: Document S1. Figures S1–S3 and Tables S1 and S5 [file mmc1.pdf]

Stem Cell Reports, Volume *20*

Supplemental Information

Embracing the heterogeneity of neural stem cells in the subventricular zone

Stefania Apostolou and Vanessa Donega

Embracing the heterogeneity of neural stem cells in the subventricular zone

Stefania Apostolou[1] and Vanessa Donega[1,2]

[1]Amsterdam UMC location Vrije Universiteit Amsterdam, department of Anatomy and Neurosciences, De Boelelaan 1117, Amsterdam, The Netherlands.
[2]Amsterdam Neuroscience, Cellular and Molecular Mechanisms, Amsterdam, the Netherlands.

Supplemental material

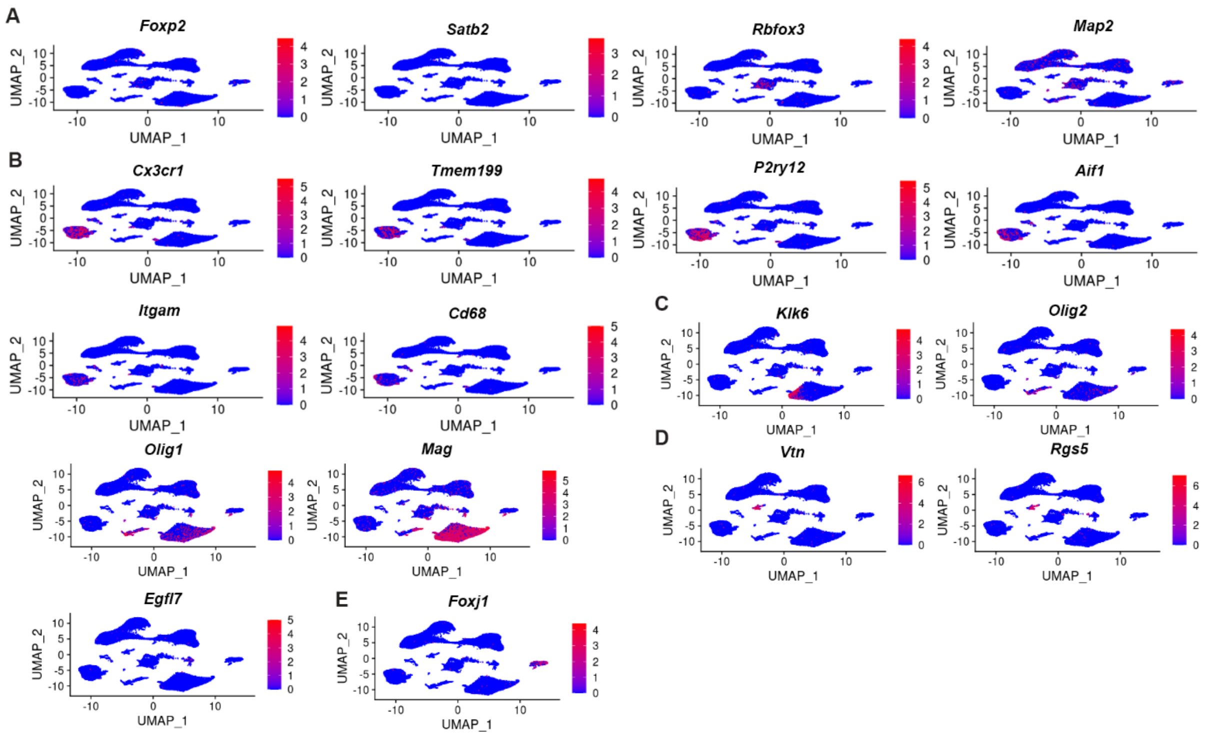

Figure S1. Identification of different cell types. A-E Feature plots for a select number of canonical markers for neurons (A), microglia (B), oligodendrocytes (C), endothelial cells (D), and ependymal cells (E).

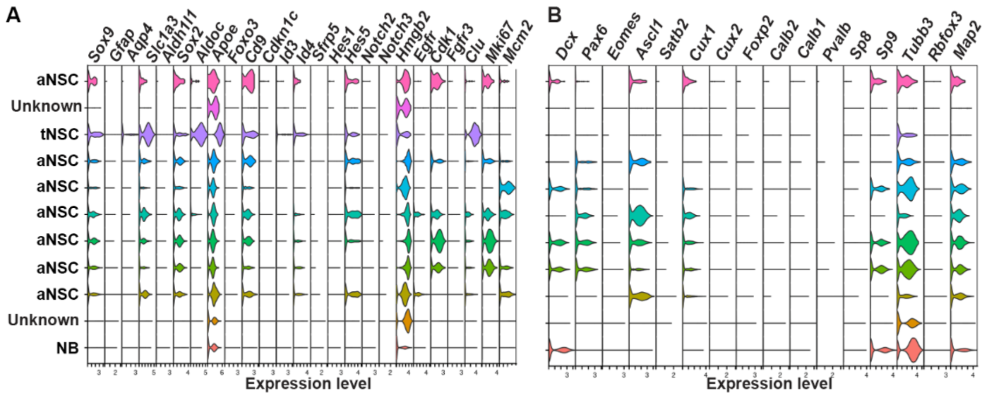

Figure S2. Gene expression in aNSC dataset. (A-B) Violin plots showing the expression of astrocyte, NSC, quiescence, and activation gene markers (A) and neuroblasts, GABAergic and glutamatergic progenitors, and neuronal gene markers (B) in the population of aNSCs.

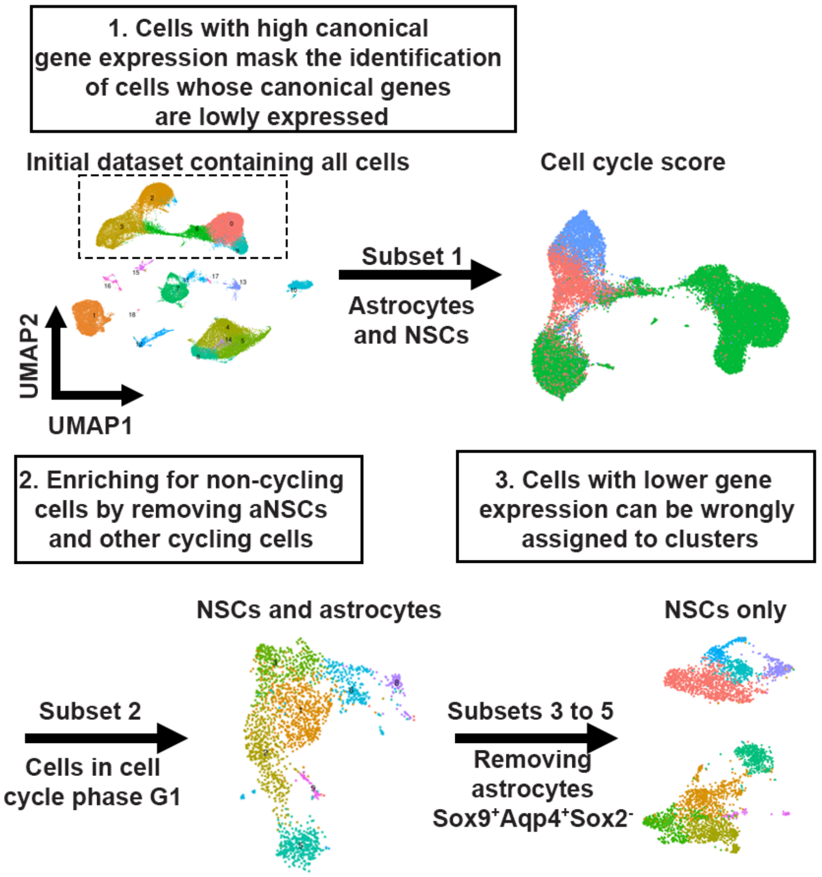

Figure S3. Schematic overview of the workflow used to subset NSCs from astrocytes. Following integration of the six datasets, astrocytes and NSCs were subsetted to remove microglia cells and other cells that are not of interest (Subset 1). Following each subsetting, ScaleData and unbiased cluster analysis were run. Cell cycle score analysis was performed using the cell cycle regression analysis pipeline from Seurat. The dataset was enriched for astrocytes and qNSCs by subsetting cells in the cell cycle phase G1, thereby removing aNSCs (Subset 2). Astrocytes were identified (Sox9$^+$Aqp4$^+$Sox2$^-$) and removed from the dataset in three subset rounds (Subsets 3 to 5). Key take home messages are depicted in the text boxes numbered 1 to 3.

Study	Sex	Age	Marker	Region SVZ
**Dulken et al., 2017**	Male	90 days	Gfap reporter mice and FACS for Prom1	Lateral
**Hamed et al., 2022**	Male and female	39 days	Sox2 reporter mouse line	Dorsal, lateral, and medial
**Kalamakis et al., 2019**	Male and female	60 days	Gfap reporter mice and FACS for Prom1	Lateral
**Mizrak et al., 2020a**	Male and female	60 days	Gfap reporter mouse line	Lateral and medial
**Mizrak et al., 2020b**	Male	60 days	Nestin reporter mouse line	Lateral and medial
**Xie et al., 2020**	Male and female	90 days	Nestin reporter mouse line	Dorsal and lateral

Table S1. Overview of the single-cell RNA sequence datasets used in this study with information on sex and age of mice, approach used to identify NSCs, and region of the SVZ that was dissociated.

Table S2. S score, and G2_M score per cell calculated with the cell cycle scoring analysis script from the Seurat pipeline. *Supplementary excel sheet.*

Table S3. List of differentially expressed genes for each of the clusters in Figure 3E. Differential gene expression was tested with Bonferroni corrected Wilcoxon rank sum test. Genes were considered significant when adjusted P-value < 0.01. Genes were considered cluster marker genes when adjusted P-value < 0.01, and it was lowly expressed in one of the two groups (pct2 < 0.5), while being expressed in more than half of the cells in the other group (pct1 > 0.5). pct = the percentage of cells where the feature gene is detected in the first or second group. *Supplementary excel sheet.*

Table S4. List of differentially expressed genes between cluster 1 and cluster 2. Differential gene expression was tested with Bonferroni corrected Wilcoxon rank sum test. Genes were considered significant when adjusted P-value < 0.01. *Supplementary excel sheet.*

Gene	qNSC	pqNSC	tNSC	aNSC	NPC/NB	Astrocyte
Aqp4	Red	Red	Grey	Blue	Grey	Red
Aldoc	Red	Red	Red	Grey	Blue	Red
Apoe3	Red	Red	Red	Red	Red	Red
Aldh1l1	Grey	Grey	Grey	Blue	Blue	Blue
*Gfap**	Grey	Blue	Blue	Blue	Grey	Blue
Slc1a3	Red	Red	Red	Grey	Grey	Red
Sox9	Red	Red	Red	Red	Grey	Red
Sox2	Red	Red	Red	Red	Grey	Blue
Hmgb2	Blue	Blue	Blue	Red	Grey	Blue
Cd9	Red	Red	Red	Red	Grey	Red
Hes1	Grey	Blue	Grey	Blue	Blue	Blue
Hes5	Red	Red	Grey	Grey	Blue	Grey
Id3	Red	Red	Red	Blue	Blue	Grey
Id4	Red	Blue	Grey	Grey	Blue	Grey
Sfrp5	Blue	Blue	Red	Blue	Blue	Blue
Notch2	Blue	Grey	Blue	Blue	Blue	Blue
Ascl1	Blue	Grey	Grey	Grey	Grey	Blue
Egfr	Blue	Grey	Grey	Grey	Grey	Blue
Fgfr3	Blue	Grey	Grey	Blue	Blue	Grey
Mcm2	Blue	Blue	Blue	Grey	Blue	Blue
Mki67	Blue	Blue	Blue	Grey	Blue	Blue
Dcx	Blue	Blue	Blue	Grey	Grey	Blue

Table S5. Summary of gene expression per NSC state and cell subtype taking individual clusters into consideration. Color coding: Red = gene is expressed; Blue = gene is not expressed; Green = mixed gene expression in clusters.
